# The dynamic history of plastome structure across aquatic subclass Alismatidae

**DOI:** 10.1186/s12870-023-04125-x

**Published:** 2023-03-04

**Authors:** Zhi-Zhong Li, Samuli Lehtonen, Jin-Ming Chen

**Affiliations:** 1 Aquatic Plant Research Center, Wuhan Botanical Garden, Chinese Academy of Sciences, Wuhan, 430074 China; 2 Center of Conservation Biology, Core Botanical Gardens, Chinese Academy of Sciences, Wuhan, 430074 China; 3 Herbarium, Biodiversity Unit, University of Turku, Turku, 20014 Finland

**Keywords:** Alismatidae, Plastomes, Inversion, Gene loss, Repeat elements

## Abstract

**Background:**

The rapidly increasing availability of complete plastomes has revealed more structural complexity in this genome under different taxonomic levels than expected, and this complexity provides important evidence for understanding the evolutionary history of angiosperms. To explore the dynamic history of plastome structure across the subclass Alismatidae, we sampled and compared 38 complete plastomes, including 17 newly assembled, representing all 12 recognized families of Alismatidae.

**Result:**

We found that plastomes size, structure, repeat elements, and gene content were highly variable across the studied species. Phylogenomic relationships among families were reconstructed and six main patterns of variation in plastome structure were revealed. Among these, the inversion from *rbcL* to *trnV-UAC* (Type I) characterized a monophyletic lineage of six families, but independently occurred also in *Caldesia grandis*. Three independent *ndh* gene loss events were uncovered across the Alismatidae. In addition, we detected a positive correlation between the number of repeat elements and the size of plastomes and IR in Alismatidae.

**Conclusion:**

In our study, *ndh* complex loss and repeat elements likely contributed to the size of plastomes in Alismatidae. Also, the *ndh* loss was more likely related to IR boundary changes than the adaptation of aquatic habits. Based on existing divergence time estimation, the Type I inversion may have occurred during the Cretaceous-Paleogene in response to the extreme paleoclimate changes. Overall, our findings will not only allow exploring the evolutionary history of Alismatidae plastome, but also provide an opportunity to test if similar environmental adaptations result in convergent restructuring in plastomes.

**Supplementary Information:**

The online version contains supplementary material available at 10.1186/s12870-023-04125-x.

## Backgroud

The subclass Alismatidae including 12 families and 500 species in ca. 54 genera, exhibits the greatest adaptive radiation of aquatic angiosperm in the world [1, 2]. Compared with the other two families (Araceae and Tofieldiaceae) of the order Alismatales, the group contains all hydrophytic life forms (submerged, floating-leaved, and emergent) and has a high concentration of water-pollinated plants [2, 3]. All marine flowering plants distributed in five families (Hydrocharitaceae, Cymodoceaceae, Ruppiaceae, Posidoniaceae, and Zosteraceae) belong to Alismatidae. Thus, Alismatidae can be considered as a key group for understanding the adaptive evolution of aquatic plants [4, 5].

Plastomes have been proven effective in solving plant phylogeny and evolution at different taxonomic levels due to their highly conserved and simple structure, and maternal inheritance [6, 7]. The recent explosion of novel plastome data has, however, revealed a large number of structural variations (SVs) in higher taxa (e.g., Malpighiales [8]; Fabaceae [9]; Araceae [10]) or microstructural changes between/within genera (e.g., *Myriophyllum* [11]; *Monochoria* [12]). Typical SVs include genomic rearrangements, gene loss, inversion and expansion/contraction of the inverted repeats (IRs) [13, 14]. For example, in the legume family (Fabaceae), many rearrangements were revealed and SVs were demonstrated to represent independent events in plastome evolution [9]. At the order level, Du et al. [15] made a plastome phylogeny of ferns and uncovered a variety of SVs supporting the deep phylogeny. In addition, plastid SVs can be related to life forms (e.g., hemiparasite [16]; phytoparasite [17]) or habitat adaptation (e.g., submerged [18]; alpine [19]). In parasitic plants, such as in some orchids [17] and Triuridaceae [20], the loss of photosynthesis-related genes has caused an extreme reduction in the size of plastomes and led to the marked reconfiguration of plastome structure. Iles et al. [21] and Peredo et al. [18] suggested that the loss of the *ndh* complex in aquatic angiosperms might explain the mechanism of reducing photooxidative stresses in submerged habitats. As well, SVs in plastomes may be associated with IR expansion/contraction, which in turn affects the plastome size [9]. The studies of Fu et al. [22, 23] revealed frequent gene loss (i.e., *ndh* complex) and plastome degradation in the genus *Gentiana*, which was suggested to parallel the diversification in this group. In Alismatidae, Ross et al. [24] found that the independent loss of the *ndh* complex in three families (Hydrocharitaceae, Cymodoceaceae, and Posidoniaceae) of Alismatidae may be associated with changes in plastome sizes [e.g., *Thalassia hemprichii* (loss of *ndh* complex; 178,261 bp) vs. *Zostera marina* (keep *ndh* complex; 143,877 bp)].

Previous studies have suggested that abundant repetitive elements, such as simple sequence repeats (SSRs), tandem repeats (TRs), and dispersed repeats, are commonly observed in rearranged plastomes and may be important factors driving the variation in plastome size [25]. Several studies have confirmed a correlation between repeat content with plastome size, for example, in Geraniaceae [26], Fabaceae [27], and Gymnosperms [28], or found that repeats promote rearrangements [29].

Until now, approximately 50 Alismatidae plastomes, representing ca. eight families and 20 genera, have been assembled and are available from GenBank (accessed on 1 January 2022). However, some genera have been extensively sampled (e.g., *Ottelia* [30]; *Hydrocharis* [31]), certain families lack accessible plastome resources. Thus, a comprehensive comparison of plastomes across Alismatidae remains scarce in the existing investigations.

In this study, we newly assembled 17 plastomes belonging to eight families in Alismatidae. We combined them with a set of existing complete plastomes to investigate the plastome structural evolution that covers all Alismatidae families, focusing on 1) exploring the pattern of SVs across Alismatidae plastomes; 2) detecting the correlations between plastome size and repetitive elements.

## Results

### Characteristics of Alismatidae plastomes

**Fig. 1:**
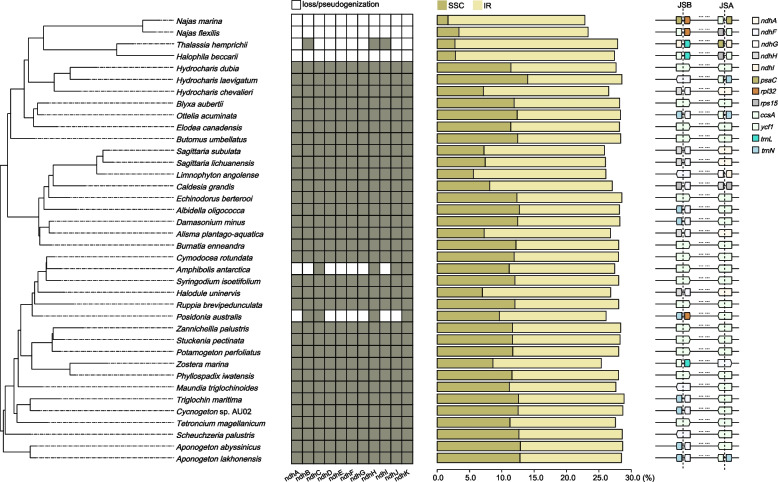
Distribution of *ndh* gene loss, size of LSC and IR, and IR boundary across the phylogenetic tree in Alismatidae

A total of 17 new plastomes representing six families were assembled and annotated here (Table S1). In combination with previously published data, the plastome size in Alismatidae displayed a size variation ranging from 143,877 bp (*Zostera marina*) to 179,007 bp (*Sagittaria lichuanensis*), with the normal quadripartite structure consisting of a large single copy (LSC, 80,881–99,125 bp), a small single copy (SSC, 2666–21,476 bp), and one pair of IRs (22,292–44,815 bp). Similarly, the GC content varied from 35.5% (*Amphibolis antarctica* and *Z. marina*) to 39.2% (*T. hemprichii*). All plastomes included 30 tRNA and four rRNA, whereas the number of PCGs varied from 68 to 79 (Table S1). This variation was explained by several gene loss events. The *ndh* complex was generally lost or pseudogenized in *Najas* and some marine species, including *T*. *hemprichii*, *Halophila beccarii, A. antarctica,* and *Posidonia australis* (Fig. 1). Also, two genes, comprised of *rps19* and *psbA*, and one *rps16* gene were lost in *Z. marina* and *H. chevalieri*, respectively.

### IR extraction/expansion and structural variations

The junctions of SSC/IRa (JSA) and SSC/IRb (JSB) were compared to assess the IR extraction/expansion among the twelve families in Alismatidae. A total of 14 junction types were found. In the most common type I, the *ycf1* was located on both the JSA and JSB. This type was found to be randomly distributed in various families (Fig. 1). The next most common types were II and III, where the JSA was located between *ndhF* and *ndhH*, and JSB was expanded into the *ndhA* with type II*.* In type II, JSA was placed in the *ndhF*-*trnN* intergenic spacer, and JSB was located in the *ycf1*. Nine more types were found independently distributed in nine genera (Fig. 1).

**Fig. 2:**
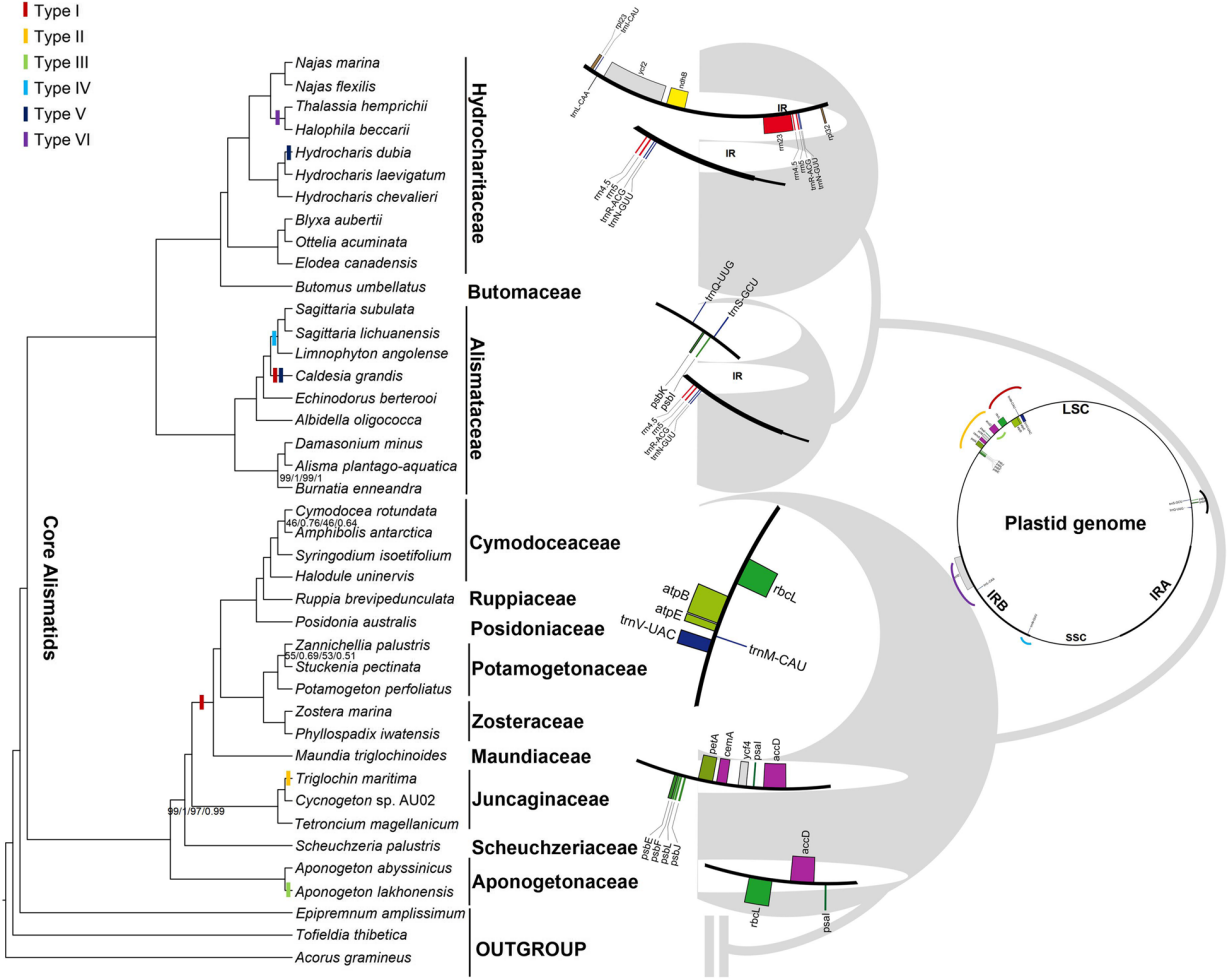
Structure variation of plastomes across the phylogeny of Alismatidae. Six types of inversion were detected: I) from *rbcL* to *trnV*-*UAC;* II) from *accD* to *psbE*; III) *accD*; IV) from trnQ-UUG to *trnS*-*GCU*; V) from *ycf2* to *trnL-CAA*; VI) *trnN-GUU*. Bootstrap supports (BS) and posterior probability (PP) are represented at nodes, except nodes with 100% BS and 1.0 PP. The first two values showed unpartitioned data, and the last two were estimated from partitioned data

Six main types (I-VI) of SVs were detected, with *A. gramineus* as a reference (Fig. 2). In types I-IV, small-scale inversions occurred in LSC regions, three of which were anchored in the boundary of the *accD* gene. In type I, a small inversion from *rbcL* to *trnV-UAC* was located in the monophyletic lineage with six families and independently in *Caldesia grandis*. Type II was detected only in *Triglochin maritima*, which displayed ~ 8 kb inversion with nine PCGs, from *accD* to *psbE*. Moreover, *accD* gene inversion, named type III, was found in *Aponogeton lakhonensis*. Type IV was found in *S. subulata*, *S. lichuanensis,* and *Limnophyton angolense* in the family Alismataceae and the region *trnQ-UUG* + *psbK* + *psbI* + *trnS-GCU* was inverted. Type V was identified in the marine lineage of Hydrocharitaceae, where *ycf2* with *trnL-CAA* was inverted in the IR region. The *trnN-GUU* gene inversion in type VI characterizes *Caldesia grandis* and *H. dubia*.

### Repeat sequences in Alismatidae plastomes

**Fig. 3:**
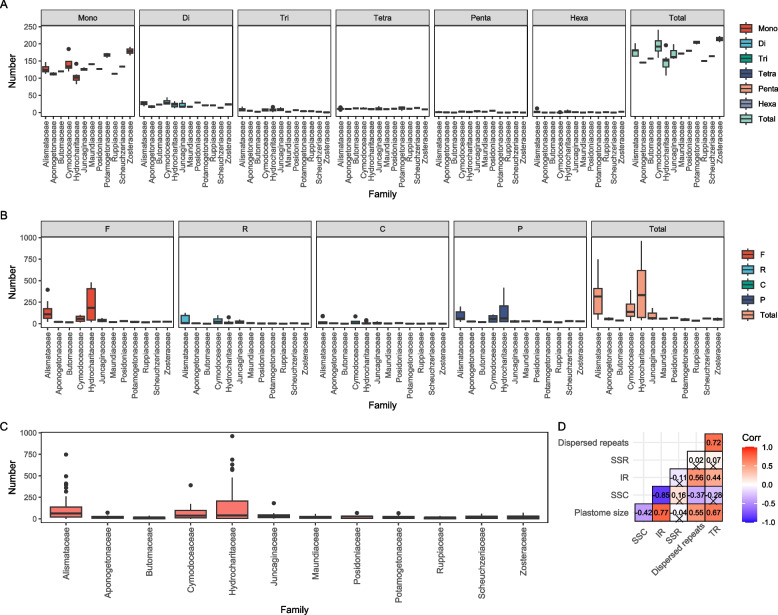
Distribution of repetitive elements and correlation analysis in Alismatidae. **A** SSR; **B** Dispersed repeats; **C** Tandem repeats; **D** correlation analysis among the repetitive elements and plastome/region size, and significant correlation (*p* < 0.05) was shown in the box without the Cross mark

Similarly to other plastome characteristics, a variable number of repeats were uncovered within Alismatidae plastomes (Fig. 3; Table S2). For SSRs, the numbers ranged from 108 (*N. flexilis*) to 241 (*A. antarctica*). The highest number of SSRs were detected in the family Zosteraceae (205–223), whereas the most variable numbers of SSRs were found in Cymodoceaceae (160–241). Out of six SSR motifs, mononucleotide repeats were most abundant in all families (83–191), followed by dinucleotide repeats (13–44). TRs exhibited a broad range, from 26 (*Elodea canadensis*) to 288 (*S. lichuanensis*). Additionally, four direction types of repeats were searched, and Hydrocharitaceae showed the most variable repeats, ranging from 40 (*Ottelia acuminata*) to 961 (*H. beccarii*). Among these repeats, forward repeats always showed the highest number (13–479) compared to the other three types. Reverse and complement types were not detected in some species, such as *E. canadensis* (Table S2).

The correlation analyses revealed a significant positive association between the size of IR and whole plastome (*R* = 0.77, *p* < 0.05; Fig. 3). In contrast, a negative correlation (*R* = -0.85, *p* < 0.05) was detected between the size of IR and SSC. The number of repeats and TRs showed a significant positive correlation with the length of IR (*R* = 0.56, *p* < 0.05; *R* = 0.44, *p* < 0.05) and the whole plastome (*R* = 0.55, *p* < 0.05; *R* = 0.67, *p* < 0.05).

### Phylogenomic analysis

Phylogenomic analyses using two partitioning strategies and tree inference methods (ML and BI) resulted in identical and strongly supported topology ( Fig. 2; Table S3). The subclass Alismatidae was monophyletic with full support (BS = 100/100; PP = 1/1) and was further divided into two major clades. The first included Alismataceae, Hydrocharitaceae, and Butomaceae, while the rest of the families formed the second clade. In clade I, Hydrocharitaceae and Butomaceae were grouped together with full support (BS = 100/100; PP = 1/1) and were resolved as the sister of Alismataceae. In clade II, Aponogetonaceae was resolved as the sister to the remaining families. Marine species, which placed in Cymodoceaceae, Ruppiaceae, and Posidoniaceae, were resolved as a monophyletic sister of Potamogetonaceae and Zosteraceae with full support (BS = 100/100; PP = 1/1).

## Discussion

Previous studies have revealed lineage-specific structural variations in plastomes of several flowering plant lineages and related them with the evolutionary history of the lineages under geological or climate change [24, 32]. For example, *accD* inversion was only found in the Asiatic species of Aponogeton (*A. lakhonensis* and *A. undulatus*) but not in the African species [33]. In Haloragaceae, Liao et al. [11] determined a 4-kb inversion in the lineage formed by *Myriophyllum* and its sister group and considered it might be associated with historical climate changes. In our study, ~ 8 kb inversion between *rbcL* and *trnV-UAC* (Type I) was found in addition to *C. grandis* in the monophyletic lineage of six families sister to the Juncaginaceae. Therefore, the inversion likely occurred after the divergence between Juncaginaceae and the other six families but before the initial diversification of these six families. According to the estimation of divergence times by Li et al. [34], this inversion took place between ca. 66 and 74 Ma, which coincides with the Cretaceous–Paleogene (K-Pg) extinction event [35]. Increasing genomic evidence has accumulated to support that angiosperms experienced a common paleopolyploidization during the K-Pg period in response to extreme climate change [36, 37]. It may be that chaotic genomic structure after polyploidization might simultaneously accelerate nucleoplasmic communication and cause non-random rearrangements in the plastome. However, until more data becomes available, this idea should be taken cautiously.

A novel finding here was that half of the inversion events (type I-III) in Alismatidae plastomes were located on or near the *accD* gene. The *accD* gene encodes the subunit of β-carboxyl transferase for Acetyl-CoA carboxylase, which plays an important role in regulating de novo fatty acid biosynthesis [38] and is essential for leaf growth and maintaining plastid compartment in tobacco [39]. Many studies have revealed a high variability of the *accD* gene across different plant lineages [40] and often anchored in rearrangement endpoints similar to what we observed here in Alismatidae (i.e., Cupressophytes [41]), which may be explained by continuous exchange with the nuclear genome.

The *ndh* genes mainly code the NADH complex, which is necessary for the electron transfer from NADH to plastoquinone in photosystem I under temperature stress [42]. We verified the previously detected three independent loss events in Hydrocharitaceae, Posidoniaceae, and Cymodoceaceae [24]. Among these, all 11 plastid *ndh* genes were completely lost in the genera of *Najas* and *Halophila*. Generally, the *ndh* gene losses have been explained by the transference of the functional *ndh* to the nuclear genome [16, 43]. Peredo et al. [18] speculated that the loss of *ndh* genes in aquatic plants might be associated with adaptation to submerged environments. However, none of the *ndh* genes were lost in other closely related submerged species, such as *O. acuminata* and *Ruppia brevipedunculata*. This indicates that the plastome evolution in aquatic angiosperm may not be as straightforward and that the gene losses are not directly related to the life forms. Thus, we prefer the hypothesis of plastid-nuclear exchange while maintaining the function of *ndh*. Interestingly, nearly half of the genes *(ndhA*,* F*,* G*,* H*,* I*) in the junction of SC/IR belong to the *ndh* complex. We observed the lowest relative size of SSC (2% in *N. marina*) and the highest relative size of IR (25% in *T. hemprichii* and *H. beccarii*) in Hydrocharitaceae, a family with complete losses of *ndh* genes (Fig. 1). Therefore, it seems reasonable to assume that the loss/pseudogenization of *ndh* genes is associated with the IR extraction/expansion, as has been suggested in Gentianaceae [22], Orobanchaceae [16], and Polygonaceae [19].

Repetitive elements have been suggested to play a key role in stabilizing the structure and size of plastomes in flowering plants [25, 26]. We found abundant repeats in plastomes throughout Alismatidae, with a significant positive correlation between the size of the plastome, IR and the number of dispersed and tandem repeats (Fig. 3). Although we did not find any distribution pattern of repeats associated with the plastome size, as in some other studies [44], it was evident that large repeats (i.e., IR) contributed to the variation of plastome size. The IR size negatively correlated with the size of SSC but positively with the size of the full plastome. Furthermore, several studies have postulated an increase in the content of repeat elements. If that is true, a similar repeat pattern might be expected in the specific lineage with the inversion of type I in contrast to outgroups (e.g., Juncaginaceae, Scheuchzeriaceae). However, we failed to find such a pattern. Thus, our results suggested that a possible explanation of illegitimate repeats might be that they are randomly generated and lost in different evolutionary lineages. Nevertheless, the effect of repeat elements on the variation of plastome structure and size is complex. Due to the limited species sampling in this study, our data is not an ideal example for understanding the dynamic change process for plastome repeats. In addition, as we have previously noted, the extremely abundant repetitive sequences in the family Alismataceae hindered the successful assembly of complete plastomes [5]. Future studies of plastome evolution in Alismatidae might benefit from long-read sequencing technologies (e.g., PacBio, Nanopore).

## Conclusion

Our study is the first to investigate the structural variation of plastomes in Alismatidae at the family level. Comparative analyses revealed high variation in plastome size, repetitive elements among all twelve families and the specific-lineage inversion from *rbcL* and *trnV*-*UAC* (Type I). Three independent *ndh* loss events were identified across the Alismatidae more likely in association with the IR extraction/expansion rather than as adaptation to certain habitats. However, we should note that before we can fully answer these questions, we need to know if these lost/pseudogenized genes are translocated in the nuclear genome instead. In addition, we detected a positive correlation between the number of repeat elements and the size of plastomes and IR in Alismatidae. In summary, our findings allowed exploration of the evolutionary history of Alismatidae plastome and also can provide an opportunity to test if similar environmental adaptations resulted in convergent restructuring in plastomes.

## Materials and methods

### Plant materials, DNA extraction, sequencing, and plastome assembly

A total of 38 species representing all 12 accepted families in Alismatidae were incorporated into this study. These included 1) 13 species belonging to five families newly sequenced in this study; 2) 21 plastomes from eight families downloaded from GenBank, 3) the sequenced data of the remaining four species, *Amphibolis antarctica* (SRR19106495), *Najas marina* (ERR5529706), *Posidonia australis* (SRR19106496) and *Zannichellia palustris* (ERR5554861), retrieved from the SRA database (Table S1). Three additional plastomes from Araceae, Tofieldiaceae, and Acoraceae were included as outgroups. The field sampling followed the ethics and legality of the local government and was permitted by the government.

Total genomic DNA extraction, DNA fragmentation and preparation of sequencing libraries of all 13 newly sampled species followed the description by Li et al. [31]. Genome skimming per sample was conducted on the Illumina HiSeq 2000 platform in Novogene (Tianjin, China), with 150 bp paired-end reads. Newly sequenced reads of 13 species and four SRA sequenced reads were filtered with Fastp v.0.20.1 [45] using default settings. Complete plastomes were de novo assembled using the software GetOrganelle v.1.7.1 [46] with default parameters. Plastid Genome Annotator (PGA [47]) and the online tool Geseq [48] were utilized for the annotation of assembled plastomes. The annotations for protein-coding genes (PCGs) were checked and adjusted manually according to published plastomes of Alismatales. All newly generated plastomes were deposited in the China National GeneBank DataBase (CNGBdb, https://db.cngb.org/; Table S1).

### Comparative plastome analysis

The plastome information, including the gene content, the proportion of coding and non-coding regions, and average gene density (genes/kb), were calculated using a custom python script. The IR extraction/expansion and structural variations (SVs) were detected using Geneious R v.11.0.5 (Biomatters, Auckland, New Zealand) with default settings.

### Characterization of SSRs and repetitive sequences

The MISA script [49] was utilized to search the SSRs with the minimal parameters set to eight repeat motifs for mononucleotides, five for dinucleotides, four for trinucleotides and three for tetranucleotide, pentanucleotide and hexanucleotide repeats. Also, four types of dispersed repeats, including Forward (F), Reverse(R), Complement (C), and Palindromic (P), were identified using the online tool REPuter [50], with settings of a Hamming distance of three and minimal repeat size of 30 bp. Tandem repeats (TRs) within plastomes were further identified using the Tandem Repeats Finder program [51] with default parameters.

### Phylogenetic reconstruction

A total of 41 plastomes, representing thirty-eight species from Alismatidae and three outgroup species (*Tofieldia thibetica*, *Epipremnum amplissimum,* and *Acorus gramineus*), was included in our phylogenetic analyses. All PCGs of each plastome were extracted and aligned using MAFFT v. 7.505 implemented in PhyloSuite v. 1.2.2 [52]. TrimAl v. 1.2 [53] was used to remove the ambiguous regions from the aligned sequence matrix with default settings before concatenating all clean alignments to a supermatrix. Phylogenetic analyses employing Maximum likelihood (ML) and Bayesian inference (BI) were conducted using unpartitioned data and data partitioned by genes. In the partitioned strategy, the best partitioning scheme was inferred using PartitionFinder v. 2.1.1 [54]. The program ModelFinder [55] was further used for searching the best substitution models. ML analysis was conducted in IQ-TREE v. 1.6.12 [56] with 5,000 rapid bootstrap replicates (BS). BI was carried on with MrBayes v. 3.2.7 [57] by running 2 × 10^7^ generations and sampling every 1000 generations. The first 20% of trees were discarded as burn-in, and the remaining trees were summarized in a majority-rule consensus tree with the Bayesian posterior probabilities (PP).

### Correlation analysis

Pearson’s correlation coefficients with significance level between the repeat (SSRs, TRs, repetitive sequences) and plastome sizes (whole genome, IR, and SSC) were conducted and visualized using the R package ‘ggcorrplot’ [58].

## Supplementary Information

**Additional file 1: Table S1.** The genome content of all plastome sequences used in this study.

**Additional file 2: Table S2.** Detailed information on repetitive elements across Alismatidae plastomes in this study.

**Additional file 3: Table S3.** The best partition scheme and models were estimated based on PartitionFinder analysis.

## Data Availability

All newly annotated plastomes in this study are available from the China. National GeneBank DataBase with CNGB-Project ID: CNP0003742 and accession numbers: N_001484957, and N_001484959 to N_001484974 (See Additional file: Table S1).

## Abbreviations

BI: Bayesian inference
bp: Base pair
BS: Bootstrap support
IR: Inverted repeat
LSC: Large single copy
ML: Maximum Likelihood
PCGs: Protein coding genes
PP: Posterior probability
rRNA: Ribosomal RNA
SSC: Small single copy
SSR: Simple sequence repeat
SVs: Structural variations
tRNA: Transfer RNA
TRs: Tandem repeats
