# Supplementary material for: The dynamic history of plastome structure across aquatic subclass Alismatidae
Source: BMC Plant Biol. 2023 Mar 4;23:125. doi: 10.1186/s12870-023-04125-x (PMC9985265; doi:10.1186/s12870-023-04125-x)
Supplement: Supplementary file 1 — Additional file 1: Table S1. The genome content of all plastome sequences used in this study. [file 12870_2023_4125_MOESM1_ESM.docx]

**Table S1. The genome content of all plastome sequences used in this study.**

| **Family** | **Name of organism** | **GenBank accession number** | **Sample site** | **Voucher No.** | **Genome size (bp)** | **LSC length (bp)** | **SSC length (bp)** | **IR length (bp)** | **Number of genes** | **Number of protein-coding genes (duplicated in IR)** | **Number of tRNA genes (duplicated in IR)** | **Number of rRNA genes (duplicated in IR)** | **Number of genes with one intron (two introns)** | **Proportion of coding to non-coding regions** | **Average gene density (genes/kb)** | **GC content (%)** |
| --- | --- | --- | --- | --- | --- | --- | --- | --- | --- | --- | --- | --- | --- | --- | --- | --- |
| Alismataceae | ***Damasonium minus*** | N_001484963 | Kew DNA bank | 37183 | 159,562 | 89,262 | 19,864 | 25,218 | 113 | 79 (6) | 30 (7) | 4 (4) | 16 (2) | 0.7 | 0.81 | 36 |
| Alismataceae | ***Albidella oligococca*** | N_001484957 | Darwin, Australia | - | 160,693 | 90,534 | 20,403 | 24,878 | 113 | 79 (6) | 30 (7) | 4 (4) | 16 (2) | 0.7 | 0.81 | 36.1 |
| Alismataceae | ***Burnatia enneandra*** | N_001484960 | Kenya | LZZ-KNE01 (HIB) | 162,097 | 90,912 | 19,747 | 25,719 | 113 | 79 (7) | 30 (7) | 4 (4) | 16 (2) | 0.7 | 0.81 | 35.8 |
| Alismataceae | ***Limnophyton angolense*** | N_001484966 | Mangango, Zambia | LZZ-lim9-1(HIB) | 175,094 | 95,593 | 9,781 | 35,860 | 113 | 79 (11) | 30 (7) | 4 (4) | 16 (2) | 0.71 | 0.77 | 37.2 |
| Alismataceae | ***Sagittaria subulata*** | N_001484970 | Wuhan (cultivated), China | Luro-CLY(HIB) | 175,651 | 97,556 | 12,711 | 32,692 | 113 | 79 (8) | 30 (7) | 4 (4) | 16 (2) | 0.69 | 0.75 | 36.9 |
| Alismataceae | *Caldesia grandis* | MH262300.2 | / | / | 168,500 | 90,985 | 13,679 | 31,918 | 113 | 79 (8) | 30 (7) | 4 (4) | 16 (2) | 0.71 | 0.78 | 37.1 |
| Alismataceae | *Sagittaria lichuanensis* | NC_029815 | / | / | 179,007 | 99,125 | 13,278 | 33,302 | 113 | 79 (9) | 30 (7) | 4 (4) | 16 (2) | 0.69 | 0.74 | 36.8 |
| Alismataceae | *Alisma plantago-aquatica* | NC_044108.1 | / | / | 167,642 | 90,004 | 12,208 | 32,715 | 113 | 79 (10) | 30 (7) | 4 (4) | 16 (2) | 0.71 | 0.8 | 35.7 |
| Alismataceae | ***Echinodorus berterooi*** | N_001484964 | Veracruz, Mexico | SL412 (TUR) | 164,862 | 91,109 | 20,349 | 26,702 | 113 | 79 (6) | 30 (7) | 4 (4) | 16 (2) | 0.69 | 0.79 | 36.1 |
| Aponogetonaceae | *Aponogeton abyssinicus* | NC_053910.1 | / | / | 154,562 | 85,785 | 19,873 | 24,452 | 113 | 79 (6) | 30 (7) | 4 (4) | 16 (2) | 0.72 | 0.84 | 36.9 |
| Aponogetonaceae | *Aponogeton lakhonensis* | NC_053912.1 | / | / | 154,860 | 86,420 | 19,804 | 24,321 | 113 | 79 (6) | 30 (7) | 4 (4) | 16 (2) | 0.72 | 0.84 | 36.9 |
| Butomaceae | *Butomus umbellatus* | NC_051949.1 | / | / | 158,107 | 88,140 | 19,695 | 25,148 | 113 | 79 (7) | 30 (7) | 4 (4) | 16 (2) | 0.71 | 0.83 | 36.8 |
| Cymodoceaceae | *Syringodium isoetifolium* | MZ325253.1 | / | / | 159,333 | 89,055 | 19,160 | 25,559 | 113 | 79 (7) | 30 (7) | 4 (4) | 16 (2) | 0.7 | 0.82 | 35.9 |
| Cymodoceaceae | ***Halodule uninervis*** | N_001484965 | Hainan, China | LZZ-HN30 (HIB) | 164,005 | 87,468 | 11,481 | 32,528 | 113 | 79 (9) | 30 (7) | 4 (4) | 16 (2) | 0.72 | 0.81 | 36.3 |
| Cymodoceaceae | ***Cymodocea rotundata*** | N_001484962 | Hainan, China | LZZ-HN31 (HIB) | 158,311 | 88,451 | 18,836 | 25,512 | 113 | 79 (7) | 30 (7) | 4 (4) | 16 (2) | 0.71 | 0.83 | 36.3 |
| Cymodoceaceae | *Amphibolis antarctica** | N_001484959 | / | / | 157,103 | 88,362 | 17,497 | 25,622 | 106 | 72 (6) | 30 (7) | 4 (4) | 13 (2) | 0.63 | 0.78 | 35.5 |
| Hydrocharitaceae | *Najas marina** | N_001484968 | / | / | 158,013 | 88,559 | 2,666 | 33,394 | 102 | 68 (8) | 30 (7) | 4 (4) | 13 (2) | 0.67 | 0.77 | 37.9 |
| Hydrocharitaceae | *Elodea canadensis* | NC_018541.1 | / | / | 156,700 | 86,194 | 17,808 | 26,349 | 113 | 79 (7) | 30 (7) | 4 (4) | 16 (2) | 0.72 | 0.84 | 37 |
| Hydrocharitaceae | *Najas flexilis* | NC_021936.1 | / | / | 156,366 | 90,572 | 5,266 | 31,207 | 102 | 68 (5) | 30 (7) | 4 (4) | 13 (2) | 0.6 | 0.75 | 38.2 |
| Hydrocharitaceae | *Thalassia hemprichii* | NC_043774.1 | / | / | 178,261 | 83,691 | 4,940 | 44,815 | 105 | 71 (20) | 30 (7) | 4 (4) | 14 (2) | 0.65 | 0.76 | 39.2 |
| Hydrocharitaceae | *Halophila beccarii* | NC_051970.1 | / | / | 168,585 | 80,881 | 4,730 | 41,487 | 102 | 68 (16) | 30 (8) | 4 (4) | 13 (2) | 0.66 | 0.77 | 38.5 |
| Hydrocharitaceae | *Blyxa aubertii* | NC_060996.1 | / | / | 158,187 | 87,799 | 18,814 | 25,798 | 113 | 79 (7) | 30 (7) | 4 (4) | 16 (2) | 0.71 | 0.83 | 36.5 |
| Hydrocharitaceae | *Ottelia acuminata* | NC_060997.1 | / | / | 156,995 | 87,477 | 19,418 | 25,050 | 113 | 79 (6) | 30 (7) | 4 (4) | 16 (2) | 0.71 | 0.83 | 36.6 |
| Hydrocharitaceae | *Hydrocharis dubia* | NC_061221.1 | / | / | 159,698 | 89,581 | 18,211 | 25,953 | 113 | 79 (5) | 30 (7) | 4 (4) | 16 (2) | 0.73 | 0.81 | 37.2 |
| Hydrocharitaceae | *Hydrocharis laevigatum* | NC_061223.1 | / | / | 153,373 | 87,313 | 21,476 | 22,292 | 113 | 79 (4) | 30 (7) | 4 (4) | 16 (2) | 0.73 | 0.83 | 37 |
| Hydrocharitaceae | *Hydrocharis chevalieri* | NC_061224.1 | / | / | 158,066 | 85,578 | 11,278 | 30,605 | 112 | 78 (7) | 30 (7) | 4 (4) | 15 (2) | 0.74 | 0.82 | 37 |
| Juncaginaceae | ***Triglochin maritima*** | N_001484973 | Wuhan (cultivated), China | LZZ-5-2 (HIB) | 155,734 | 85,289 | 19,571 | 25,437 | 113 | 79 (5) | 30 (7) | 4 (4) | 16 (2) | 0.7 | 0.83 | 36.5 |
| Juncaginaceae | ***Cycnogeton*** sp. AU02 | N_001484961 | Wuhan (cultivated), China | LZZ-AU02(HIB) | 156,942 | 86,498 | 19,666 | 25,389 | 113 | 79 (5) | 30 (7) | 4 (4) | 16 (2) | 0.7 | 0.82 | 36.9 |
| Juncaginaceae | ***Tetroncium magellanicum*** | N_001484972 | Kew DNA bank | S12196 | 162,206 | 91,024 | 18,234 | 26,474 | 113 | 79 (7) | 30 (7) | 4 (4) | 16 (2) | 0.7 | 0.81 | 36 |
| Maundiaceae | ***Maundia triglochinoides*** | N_001484967 | New South Wales, Australia | - | 157,825 | 88,278 | 17,603 | 25,972 | 113 | 79 (7) | 30 (7) | 4 (4) | 16 (2) | 0.72 | 0.83 | 36.2 |
| Posidoniaceae | *Posidonia australis** | N_001484969 | / | / | 152,983 | 87,784 | 14,697 | 25,251 | 106 | 72 (6) | 30 (8) | 4 (4) | 15 (2) | 0.68 | 0.81 | 37 |
| Potamogetonaceae | *Zannichellia palustris** | N_001484974 | / | / | 154,953 | 85,127 | 18,058 | 25,884 | 113 | 79 (7) | 30 (8) | 4 (4) | 16 (2) | 0.72 | 0.85 | 35.9 |
| Potamogetonaceae | *Potamogeton perfoliatus* | NC_029814.1 | / | / | 156,226 | 86,764 | 18,238 | 25,612 | 113 | 79 (7) | 30 (7) | 4 (4) | 16 (2) | 0.72 | 0.84 | 36.5 |
| Potamogetonaceae | *Stuckenia pectinata* | NC_057253.1 | / | / | 156,669 | 86,285 | 18,236 | 26,074 | 113 | 79 (7) | 30 (8) | 4 (4) | 16 (2) | 0.72 | 0.84 | 36.5 |
| Ruppiaceae | *Ruppia brevipedunculata* | NC_051974.1 | / | / | 158,943 | 88,857 | 19,130 | 25,481 | 113 | 79 (7) | 30 (7) | 4 (4) | 16 (2) | 0.7 | 0.82 | 35.8 |
| Scheuchzeriaceae | ***Scheuchzeria palustris*** | N_001484971 | Wuhan (cultivated), China | LZZ-BZC (HIB) | 158,573 | 87,881 | 20,070 | 25,311 | 113 | 79 (6) | 30 (7) | 4 (4) | 16 (2) | 0.71 | 0.82 | 37.3 |
| Zosteraceae | *Zostera marina* | NC_036014.1 | / | / | 143,877 | 83,247 | 12,398 | 24,116 | 111 | 77 (7) | 30 (8) | 4 (4) | 16 (2) | 0.71 | 0.9 | 35.5 |
| Zosteraceae | *Phyllospadix iwatensis* | NC_058622.1 | / | / | 152,726 | 84,767 | 17,639 | 25,168 | 113 | 79 (7) | 30 (8) | 4 (4) | 16 (2) | 0.72 | 0.86 | 36.2 |
| Tofieldiaceae | *Tofieldia thibetica* | NC_029813.1 | / | / | 155,512 | 84,584 | 18,150 | 26,389 | 113 | 79 (7) | 30 (7) | 4 (4) | 16 (2) | 0.72 | 0.84 | 37.4 |
| Araceae | *Epipremnum amplissimum* | NC_047232.1 | / | / | 163,335 | 89,714 | 22,053 | 25,784 | 113 | 79 (6) | 30 (7) | 4 (4) | 16 (2) | 0.69 | 0.8 | 36.1 |
| Acoraceae | *Acorus gramineus* | NC_026299 | / | / | 152,849 | 82,977 | 18,228 | 25,822 | 112 | 78 (6) | 30 (8) | 4 (4) | 15 (2) | 0.71 | 0.85 | 38.7 |

**Note:** HIB- Wuhan Institute of Botany; TUR-University of Turku
