# Supplementary material for: The dynamic history of plastome structure across aquatic subclass Alismatidae
Source: BMC Plant Biol. 2023 Mar 4;23:125. doi: 10.1186/s12870-023-04125-x (PMC9985265; doi:10.1186/s12870-023-04125-x)
Supplement: Supplementary file 2 — Additional file 2: Table S2. Detailed information on repetitive elements across Alismatidae plastomes in this study. [file 12870_2023_4125_MOESM2_ESM.docx]

**Table S2. Detailed information on repetitive elements across Alismatidae plastomes in this study**

| **Family** | **Name of organism** | **SSR** | | | | | | |  | **Dispersed repeats** | | | | |  | **Tandem repeats** |
| --- | --- | --- | --- | --- | --- | --- | --- | --- | --- | --- | --- | --- | --- | --- | --- | --- |
|  |  | **Mono** | **Di** | **Tri** | **Tetra** | **Penta** | **Hexa** | **Total** |  | **P** | **R** | **C** | **F** | **Total** |  |  |
| Alismataceae | *Damasonium minus* | 136 | 27 | 5 | 11 | 1 | 2 | 182 |  | 21 | 5 | 1 | 21 | 48 |  | 65 |
| Alismataceae | *Albidella oligococca* | 119 | 18 | 16 | 10 | 0 | 3 | 166 |  | 61 | 109 | 20 | 171 | 361 |  | 122 |
| Alismataceae | *Burnatia enneandra* | 133 | 31 | 7 | 11 | 0 | 0 | 182 |  | 26 | 3 | 2 | 26 | 57 |  | 111 |
| Alismataceae | *Limnophyton angolense* | 124 | 23 | 8 | 8 | 1 | 0 | 164 |  | 51 | 8 | 5 | 66 | 130 |  | 110 |
| Alismataceae | *Sagittaria subulata* | 130 | 22 | 11 | 11 | 4 | 3 | 181 |  | 199 | 26 | 9 | 261 | 495 |  | 191 |
| Alismataceae | *Caldesia grandis* | 116 | 23 | 10 | 10 | 0 | 4 | 163 |  | 49 | 1 | 1 | 63 | 114 |  | 90 |
| Alismataceae | *Sagittaria lichuanensis* | 120 | 29 | 17 | 11 | 2 | 12 | 191 |  | 138 | 125 | 90 | 394 | 747 |  | 288 |
| Alismataceae | *Alisma plantago-aquatica* | 147 | 32 | 5 | 15 | 3 | 0 | 202 |  | 119 | 95 | 86 | 110 | 410 |  | 76 |
| Alismataceae | *Echinodorus berterooi* | 113 | 32 | 5 | 9 | 2 | 2 | 163 |  | 142 | 1 | 0 | 174 | 317 |  | 167 |
| Aponogetonaceae | *Aponogeton abyssinicus* | 108 | 21 | 5 | 9 | 1 | 0 | 144 |  | 21 | 1 | 0 | 17 | 39 |  | 40 |
| Aponogetonaceae | *Aponogeton lakhonensis* | 117 | 13 | 5 | 12 | 0 | 0 | 147 |  | 30 | 7 | 9 | 26 | 72 |  | 54 |
| Butomaceae | *Butomus umbellatus* | 120 | 23 | 2 | 12 | 0 | 0 | 157 |  | 19 | 0 | 1 | 17 | 37 |  | 33 |
| Cymodoceaceae | *Syringodium isoetifolium* | 137 | 44 | 5 | 10 | 1 | 1 | 198 |  | 106 | 98 | 85 | 101 | 390 |  | 132 |
| Cymodoceaceae | *Halodule uninervis* | 120 | 24 | 3 | 11 | 2 | 0 | 160 |  | 90 | 2 | 0 | 81 | 173 |  | 82 |
| Cymodoceaceae | *Cymodocea rotundata* | 132 | 25 | 11 | 15 | 3 | 0 | 186 |  | 14 | 2 | 0 | 16 | 32 |  | 86 |
| Cymodoceaceae | *Amphibolis_antarctica* | 185 | 31 | 10 | 12 | 3 | 0 | 241 |  | 21 | 43 | 8 | 31 | 103 |  | 65 |
| Hydrocharitaceae | *Najas marina* | 94 | 22 | 1 | 11 | 0 | 1 | 129 |  | 39 | 6 | 5 | 44 | 94 |  | 98 |
| Hydrocharitaceae | *Elodea canadensis* | 91 | 15 | 6 | 9 | 1 | 2 | 124 |  | 22 | 0 | 0 | 49 | 71 |  | 26 |
| Hydrocharitaceae | *Najas flexilis* | 83 | 13 | 1 | 10 | 1 | 0 | 108 |  | 30 | 5 | 1 | 42 | 78 |  | 110 |
| Hydrocharitaceae | *Thalassia hemprichii* | 106 | 16 | 16 | 9 | 3 | 1 | 151 |  | 150 | 74 | 41 | 317 | 582 |  | 134 |
| Hydrocharitaceae | *Halophila beccarii* | 142 | 20 | 12 | 12 | 4 | 6 | 196 |  | 418 | 34 | 30 | 479 | 961 |  | 160 |
| Hydrocharitaceae | *Blyxa aubertii* | 111 | 25 | 7 | 11 | 2 | 0 | 156 |  | 20 | 1 | 0 | 27 | 48 |  | 43 |
| Hydrocharitaceae | *Ottelia acuminata* | 108 | 33 | 8 | 11 | 1 | 0 | 161 |  | 18 | 3 | 2 | 17 | 40 |  | 45 |
| Hydrocharitaceae | *Hydrocharis dubia* | 94 | 27 | 8 | 9 | 0 | 5 | 143 |  | 226 | 21 | 11 | 430 | 688 |  | 198 |
| Hydrocharitaceae | *Hydrocharis laevigatum* | 104 | 33 | 5 | 8 | 0 | 4 | 154 |  | 88 | 2 | 2 | 477 | 569 |  | 101 |
| Hydrocharitaceae | *Hydrocharis chevalieri* | 100 | 24 | 10 | 7 | 3 | 8 | 152 |  | 291 | 0 | 1 | 339 | 631 |  | 78 |
| Juncaginaceae | *Triglochin maritima* | 131 | 17 | 7 | 7 | 0 | 0 | 162 |  | 23 | 4 | 1 | 18 | 46 |  | 47 |
| Juncaginaceae | *Cycnogeton* sp. AU02 | 122 | 16 | 4 | 9 | 4 | 2 | 157 |  | 19 | 12 | 3 | 32 | 66 |  | 67 |
| Juncaginaceae | *Tetroncium magellanicum* | 125 | 36 | 15 | 18 | 4 | 1 | 199 |  | 42 | 48 | 27 | 65 | 182 |  | 181 |
| Maundiaceae | *Maundia triglochinoides* | 141 | 17 | 2 | 10 | 2 | 0 | 172 |  | 28 | 7 | 3 | 19 | 57 |  | 70 |
| Posidoniaceae | *Posidonia australis* | 127 | 29 | 7 | 11 | 5 | 1 | 180 |  | 29 | 3 | 6 | 30 | 68 |  | 67 |
| Potamogetonaceae | *Zannichellia palustris* | 173 | 23 | 4 | 5 | 0 | 0 | 205 |  | 21 | 7 | 6 | 32 | 66 |  | 108 |
| Potamogetonaceae | *Potamogeton perfoliatus* | 159 | 21 | 3 | 16 | 0 | 0 | 199 |  | 22 | 3 | 0 | 19 | 44 |  | 43 |
| Potamogetonaceae | *Stuckenia pectinata* | 169 | 21 | 4 | 15 | 0 | 0 | 209 |  | 20 | 1 | 0 | 16 | 37 |  | 45 |
| Ruppiaceae | *Ruppia brevipedunculata* | 113 | 21 | 4 | 11 | 0 | 1 | 150 |  | 17 | 1 | 0 | 16 | 34 |  | 37 |
| Scheuchzeriaceae | *Scheuchzeria palustris* | 134 | 14 | 2 | 13 | 1 | 0 | 164 |  | 31 | 4 | 3 | 22 | 60 |  | 59 |
| Zosteraceae | *Zostera marina* | 191 | 21 | 0 | 9 | 0 | 2 | 223 |  | 20 | 1 | 0 | 13 | 34 |  | 36 |
| Zosteraceae | *Phyllospadix iwatensis* | 167 | 26 | 1 | 9 | 0 | 2 | 205 |  | 37 | 1 | 0 | 32 | 70 |  | 46 |
