# Supplementary material for: The dynamic history of plastome structure across aquatic subclass Alismatidae
Source: BMC Plant Biol. 2023 Mar 4;23:125. doi: 10.1186/s12870-023-04125-x (PMC9985265; doi:10.1186/s12870-023-04125-x)
Supplement: Supplementary file 3 — Additional file 3: Table S3. The best partition scheme and models were estimated based on PartitionFinder analysis. [file 12870_2023_4125_MOESM3_ESM.docx]

**Table S3. The best partition scheme and models were estimated based on PartitionFinder analysis.**

| **Subset** | **Best Model** | **# sites** | **Partition names** |
| --- | --- | --- | --- |
| 1 | GTR+I+G | 6327 | *rpl36*, *atpE*, *rpoC1*, *ycf4*, *rpoB* |
| 2 | GTR+I+G | 813 | *rpl2* |
| 3 | GTR+I+G | 4122 | *rpoC2* |
| 4 | GTR+I+G | 711 | *rps2* |
| 5 | GTR+G | 6906 | *ycf2* |
| 6 | GTR+I+G | 657 | *rps3* |
| 7 | GTR+I+G | 639 | *ycf3*, *psbN* |
| 8 | GTR+G | 968 | *rpl14*, *rps4* |
| 9 | GTR+G | 750 | *rpl23*, *rps7* |
| 10 | GTR+I+G | 1617 | *rps8*, *rpl33*, *rpoA* |
| 11 | GTR+I+G | 414 | *rps11* |
| 12 | GTR+I+G | 372 | *rps12* |
| 13 | GTR+I+G | 849 | *rps14*, *atpF* |
| 14 | GTR+G | 633 | *rps15*, *rpl22* |
| 15 | GTR+I+G | 1137 | *rpl16*, *ndhK* |
| 16 | GTR+G | 243 | *rps16* |
| 17 | GTR+G | 515 | *rps18* |
| 18 | GTR+G | 276 | *rps19* |
| 19 | GTR+G | 351 | *rpl20* |
| 20 | GTR+I+G | 162 | *rpl32* |
| 21 | GTR+I+G | 1471 | *accD* |
| 22 | GTR+I+G | 2418 | *atpA*, *petA* |
| 23 | GTR+I+G | 1470 | *atpB* |
| 24 | GTR+G | 351 | *atpH*, *psbM* |
| 25 | GTR+I+G | 735 | *atpI* |
| 26 | GTR+I+G | 963 | *ccsA* |
| 27 | GTR+I+G | 675 | *cemA* |
| 28 | GTR+G | 834 | *clpP*, *infA* |
| 29 | GTR+I+G | 1520 | *matK* |
| 30 | GTR+I+G | 3144 | *ndhA*, *ndhG*, *ndhD* |
| 31 | GTR+I+G | 1386 | *ndhB* |
| 32 | GTR+I+G | 543 | *ndhC*, *psaI*, *petL* |
| 33 | GTR+I+G | 1094 | *psbI*, *ndhE*, *psaJ*, *psbH*, *ndhJ* |
| 34 | GTR+I+G | 2187 | *ndhF* |
| 35 | GTR+I+G | 1713 | *ndhI*, *ndhH* |
| 36 | GTR+I+G | 5505 | *psaA*, *psbC*, *petB*, *psbD*, *psbJ* |
| 37 | GTR+I+G | 752 | *petD*, *psaC* |
| 38 | GTR+I+G | 513 | *petN*, *psbZ*, *psbF*, *petG* |
| 39 | GTR+I+G | 2205 | *psaB* |
| 40 | GTR+I+G | 1062 | *psbA* |
| 41 | GTR+I+G | 1518 | *psbB* |
| 42 | GTR+I+G | 369 | *psbL*,*psbE* |
| 43 | GTR+G | 150 | *psbK* |
| 44 | GTR+I+G | 102 | *psbT* |
| 45 | GTR+I+G | 1431 | *rbcL* |
| 46 | GTR+I+G | 3767 | *ycf1* |
